# Matrix Metalloproteinases Inhibition by Doxycycline Rescues Extracellular Matrix Organization and Partly Reverts Myofibroblast Differentiation in Hypermobile Ehlers-Danlos Syndrome Dermal Fibroblasts: A Potential Therapeutic Target?

**DOI:** 10.3390/cells10113236

**Published:** 2021-11-19

**Authors:** Nicola Chiarelli, Nicoletta Zoppi, Marina Venturini, Daniele Capitanio, Cecilia Gelfi, Marco Ritelli, Marina Colombi

**Affiliations:** 1 Division of Biology and Genetics, Department of Molecular and Translational Medicine, University of Brescia, 25121 Brescia, Italy; nicola.chiarelli@unibs.it (N.C.); nicoletta.zoppi@unibs.it (N.Z.); marco.ritelli@unibs.it (M.R.); 2 Division of Dermatology, Department of Clinical and Experimental Sciences, Spedali Civili University Hospital, 25121 Brescia, Italy; marina.venturini@unibs.it; 3 Department of Biomedical Sciences for Health, University of Milan, 20090 Milan, Italy; daniele.capitanio@unimi.it (D.C.); cecilia.gelfi@unimi.it (C.G.); 4 IRCCS Istituto Ortopedico Galeazzi, 20161 Milan, Italy

**Keywords:** doxycycline, extracellular matrix, hypermobile Ehlers-Danlos syndrome, matrix metalloproteinases, myofibroblasts, secretome

## Abstract

Hypermobile Ehlers-Danlos syndrome (hEDS) is the most frequent type of EDS and is characterized by generalized joint hypermobility and musculoskeletal manifestations which are associated with chronic pain, and mild skin involvement along with the presence of more than a few comorbid conditions. Despite numerous research efforts, no causative gene(s) or validated biomarkers have been identified and insights into the disease-causing mechanisms remain scarce. Variability in the spectrum and severity of symptoms and progression of hEDS patients’ phenotype likely depend on a combination of age, gender, lifestyle, and the probable multitude of genes involved in hEDS. However, considering the clinical overlap with other EDS forms, which lead to abnormalities in extracellular matrix (ECM), it is plausible that the mechanisms underlying hEDS pathogenesis also affect the ECM to a certain extent. Herein, we performed a series of in vitro studies on the secretome of hEDS dermal fibroblasts that revealed a matrix metalloproteinases (MMPs) dysfunction as one of the major disease drivers by causing a detrimental feedback loop of excessive ECM degradation coupled with myofibroblast differentiation. We demonstrated that doxycycline-mediated inhibition of MMPs rescues in hEDS cells a control-like ECM organization and induces a partial reversal of their myofibroblast-like features, thus offering encouraging clues for translational studies confirming MMPs as a potential therapeutic target in hEDS with the expectation to improve patients’ quality of life and alleviate their disabilities.

## 1. Introduction

Hypermobile Ehlers-Danlos syndrome (hEDS) is probably the most common heritable connective tissue disorder (HCTD), but it remains without defined molecular bases. It is dominated by marked variable phenotype and high rate of chronic disability and is only diagnosed clinically, since no specific biomarker is currently available [1]. The last EDS nosology defined a set of strict criteria for hEDS that is diagnosed due to the presence of generalized joint hypermobility (JHM), two or more items among systemic involvement, positive family history, and musculoskeletal involvement, and exclusion of other hereditary and acquired CTDs [2]. Clinical diagnosis of hEDS remains challenging especially when considering the wide clinical variability and high incidence of several comorbidities not included in the current diagnostic criteria, such as gastrointestinal dysfunctions, early osteoarthritis (OA), recurrent soft-tissue injuries, orthostatic intolerance/tachycardia syndrome, and neurological and psychological issues such as chronic fatigue, depression, anxiety, mood disorders, impaired memory and concentration, sleep disturbance, headaches, and migraines [2].

We previously established that hEDS patient-derived dermal fibroblasts show a compromised deposition of many extracellular matrix (ECM) structural constituents including collagens (COLLs), fibronectin (FN), and tenascins (TNs), along with the lack of organization of the COLLs- and FN-specific receptors, i.e., the α2β1 and α5β1 integrins, respectively [3]. Patients’ cells also exhibit some cellular features typical of myofibroblasts, such as organization of cadherin-11 and alpha smooth muscle actin (α-SMA) stress fibers, high secreted levels of the ECM-degrading matrix metalloproteinase MMP9, along with the expression of the alternative FN receptor αvβ3 integrin, which triggers a signaling pathway involving the integrin-linked kinase (ILK) and the transcription factor Snail1/Slug sustaining this myofibroblast-like phenotype [4]. Proteome profiling of patients’ cells provided further insights into disease mechanisms by revealing protein changes essential to actin cytoskeleton dynamics and metabolic/mitochondrial functions [5]. Intriguingly, control fibroblasts treated with hEDS cells-derived conditioned media (hEDS-CM) acquire the pathological cellular phenotype, indicating that patients’ cells secrete key factors promoting ECM disarray and myofibroblast differentiation.

Myofibroblasts, which play important roles in modulating the cell and tissue milieu through the secretion of many matrisome-related proteins [6], are also recognized to exacerbate disease processes of different human conditions, as their aberrant/persistent activation causes an unbalanced ratio of secreted structural proteins (e.g., COLLs and FN) and enzymes involved in ECM protein turnover (e.g., MMPs and their inhibitors TIMPs) [7,8,9,10]. In vitro, myofibroblasts release in the CM factors that are involved in intercellular communication with significant biological effects [11]. Indeed, the secretome profiles of different human cell types identified over a thousand non-redundant proteins in CM and revealed that many of them were secreted via non-canonical pathways [12], highlighting the pivotal role of the shedding process and extracellular vesicles (EVs) in cellular communication [13]. Through cell-cell communication, the secretome maintains an appropriate microenvironment for ECM and tissue homeostasis and immune mechanisms [14,15]. Remarkably, most secreted proteins identified in the culture media can also be discovered in the proteomes of different human biofluids [12]. Early evidence indicated that in different human pathologies the cellular dysfunction of different cell types including dermal fibroblasts leads to an aberrant secretome that reprograms the microenvironment and induces myofibroblast differentiation [15,16]. Hence, in pathologic conditions the secretome changes may serve to disclose disease-specific biomarkers with a possible therapeutic relevance [14,17].

The present study was designed to define the hEDS-CM protein content and its biological effect, in the absence of any disease-causing variants, diagnostic biomarkers, and specific therapies for hEDS treatment, by performing biochemical analyses and secretome profiling, which revealed MMPs dysfunction as a major driver of the molecular mechanisms underlying the disease pathogenesis.

## 2. Materials and Methods

### 2.1. Ethical Compliance

This study was approved by the local Ethical Committee (Ethical Committee Protocol Number NP3151, Comitato Etico di Brescia, ASST degli Spedali Civili, Brescia). Six adult hEDS females and six sex- and age-matched healthy individuals were evaluated at the specialized outpatient clinic for HCTDs and EDS of the University Hospital Spedali Civili of Brescia. All recruited patients fulfilled the strict clinical criteria for an hEDS diagnosis according to the 2017 EDS nosology [1]. Demographic information (i.e., age at last clinical evaluation, other affected family members) and complete clinical features by single patient including numerous multisystemic signs/symptoms not comprised in the current nosology are summarized in Table S1.

### 2.2. Cell Cultures and Preparation of Conditioned Media (CM)

Skin biopsies derived from hEDS patients and unrelated healthy donors were obtained by standard procedures, as previously described [5]. Cells were grown between 1st and 4th in vitro passage at 37 °C in a 5% CO_2_ atmosphere in Earle’s Modified Eagle Medium (MEM) supplemented with 2 mM L-glutamine, 10% FBS, and 100 µg/mL penicillin and streptomycin (complete MEM). CM were recovered from different control and hEDS cell strains grown for 72 h in complete MEM and centrifuged at 3500× *g* for 7 min to eliminate cell debris and apoptotic bodies; CM from six control (C-CM) and patient (hEDS-CM) cell cultures were equally pooled and stored at −80 °C prior to further experiments.

For secretome analysis, confluent control and hEDS cell cultures were washed and maintained in a serum-free medium for 24 h to decrease sample complexity and increase reproducibility, allowing the detection of low abundance proteins. Subsequently, CM were centrifuged as above and stored at −80 °C prior further analysis. 15 mL of CM of each sample were concentrated using centrifugal filtration units with 5 kDa molecular weight cut-off membranes (Agilent Technologies, Cernusco sul Naviglio, Italy). Proteins were then washed three times with 50 mM ammonium bicarbonate (AMBIC) and resuspended in 200 µL AMBIC 50 mM and 0.1% Rapigest SF surfactant (Waters, Milford, MA, USA). After sonication in a water bath for 15 min, protein concentrations were determined by Pierce bicinchoninic acid (BCA) protein assay (Thermo Fisher Scientific, Rodano, Italy).

### 2.3. Immunofluorescence Microscopy (IF)

Control and hEDS cells were seeded on glass coverslips and grown in the presence of complete MEM, C-CM, or hEDS-CM up to 10 days and replenishing culture media every 48 h. To study the assembly of COLLI-ECM, cells were grown in the presence of 50 μM ascorbic acid (AA) refed every 24 h. IF analyses were performed every 48 h fixing cells in cold methanol and immunoreacting for 1 h at room temperature (R.T.) with 1:100 rabbit anti-FN polyclonal antibody (Ab) (Sigma Aldrich, #F3648, Milan, Italy) and 1 μg/mL mouse anti-TN monoclonal Ab (mAb) (Sigma Chemicals, clone BC-24, Milan, Italy), recognizing respectively all human isoforms of FN and TN, with 1:100 goat anti-COLLI Ab (Millipore-Chemicon Int., #AB758, Milan, Italy), and with 2 μg/mL anti-α-SMA mAb (Sigma Chemicals, clone 1A4, Milan, Italy). Expression and organization of the αvβ3 integrin was investigated fixing cells in 3% PFA/10 mM sucrose for 8 min, permeabilizing with 0.1% Triton X-100 for 1 min and 45 s, and immunoreacting for 1 h with 4 μg/mL mouse anti-αvβ3 integrin mAb (Millipore-Chemicon Int., clone LM609, Milan, Italy) diluted in 1% BSA. The Snail1/Slug distribution was analyzed fixing cells in 3.7% PFA/60 mM sucrose, blocking 30 min at R.T. with 0.1% non-fat milk/0.1% Tween 20/PBS 1×, and immunoreacting O.N. at +4 °C with 1:500 rabbit anti-Snail1/Slug Ab (Abcam, #ab180714, Milan, Italy) diluted in 0.1% Tween 20/PBS 1×. Once washed, cells were incubated for 1 h with anti-mouse or anti-rabbit secondary Abs conjugated to Alexa Fluor^®^ 594 and 488, respectively, or with rhodamine-conjugated anti-goat IgG (Calbiochem #T7028, Milan, Italy). IF signals were acquired by a CCD black-and-white TV camera (SensiCam-PCO Computer Optics GmbH, Kelheim, Germany) mounted on a Zeiss fluorescence Axiovert microscope and digitalized by Image Pro Plus software version 7.0.

### 2.4. Western Blotting (WB)

To analyze FN, COLLI, and TNs secreted in the culture media by WB, the protein concentration of the pooled C-CM and hEDS-CM was determined using detergent compatible Bio-Rad Dc Protein Assay (Sigma Aldrich, #1001-491004, Milan, Italy). 80 μg of total control and hEDS proteins were loaded in triplicate and separated in reducing conditions by electrophoresis using 8% SDS-PAGE. After nitrocellulose sheet transfer, membranes were blocked O.N. at 37 °C in 5% non-fat dry milk/TBS-0.1% Tween 20 (TBS-T) and immunoreacted for 3 h at R.T. with 1:1000 rabbit anti-FN Ab (Sigma Aldrich, #F3648, Milan, Italy), 1 μg/mL anti-TN mAb (Sigma Chemicals, clone BC-24, Milan, Italy), and 1:500 goat anti-COLLI Ab (Millipore-Chemicon Int., #AB758, Milan, Italy) diluted in 5% milk/TBS-T. After washing in TBS-T, membranes were incubated for 3 h at R.T. with HRP-conjugated anti-rabbit, anti-mouse IgG (Sigma Chemicals, #A8275 and #A5906, respectively, Milan, Italy), and anti-goat IgGs (Merck Millipore #401515, Milan, Italy), diluted 1:1000 in 5% milk/TBS-T, and chemiluminescent signals developed using the ECL method (Pierce, Thermo Fisher Scientific, Rodano, Italy).

### 2.5. Label-Free Liquid Chromatography with Tandem Mass Spectrometry (LC–MS/MS)

CM samples from control and hEDS cells were analyzed by differential proteomics. Protein extracts (80 µg) were in-solution reduced for 45 min in 5 mM dithiothreitol (DTT) at 60 °C, carbamidomethylated for 45 min in 15 mM iodoacetamide, and digested with mass spectrometry grade trypsin gold (Promega, Madison, WI, USA) for 16 h at 37 °C using a protein:trypsin ratio of 50:1. After acidification with trifluoracetic acid and desalting on C18 tips (Zip-Tip C18 micro, Merck Millipore, Milan, Italy), peptide samples were vacuum concentrated, reconstituted in HPLC buffer A (0.1% formic acid) and separated on a Dionex UltiMate 3000 HPLC System with an Easy Spray PepMap RSLC C18 column (250 mm, internal diameter of 75 µm) (Thermo Fisher Scientific, Rodano, Italy). A five steps acetonitrile was adopted on the samples: (ACN)/formic acid gradient (5% ACN in 0.1% formic acid for 5 min, 5–35% ACN in 0.1% formic acid for 139 min, 35–60% ACN in 0.1% formic acid for 40 min, 60–100% ACN for 1 min, 100% ACN for 10 min, at a flow rate of 0.3 µL/min), and samples were electrosprayed into an Orbitrap Tribrid Fusion (Thermo Fisher Scientific, Rodano, Italy). The LTQ-Orbitrap was operated in a positive mode in data-dependent acquisition mode to automatically alternate between a full scan (350–2000 m/z) in the Orbitrap (at resolution 60000, AGC target 1000000) and subsequent CID MS/MS in the linear ion trap of the 20 most intense peaks from full scan (normalized collision energy of 35%, 10 ms activation). Isolation window: 3 Da, unassigned charge states: rejected, charge state 1: rejected, charge states 2+, 3+, 4+: not rejected; dynamic exclusion enabled (60 s, exclusion list size: 200). Mass spectra were analyzed using MaxQuant software (Max Planck Institute of Biochemistry, Munich, Germany, version 1.6.3.3). The initial maximum allowed mass deviation was set to 6 ppm for monoisotopic precursor ions and 0.5 Da for MS/MS peaks. Enzyme specificity was set to trypsin/P, and a maximum of two missed cleavages was allowed. Carbamidomethylation was set as a fixed modification, while N-terminal acetylation and methionine oxidation were set as variable modifications. The spectra were searched by the Andromeda search engine against the *Homo sapiens* Uniprot UP000005640 sequence database (77,027 proteins, release 29 January 2021). Protein identification required at least one unique or razor peptide per protein group. Quantification in MaxQuant was performed using the built-in extracted ion chromatogram (XIC)-based label-free quantification (LFQ) algorithm using fast LFQ. The required FDR was set to 1% at the peptide, 1% at the protein and 1% at the site-modification level, and the minimum required peptide length was set to 7 amino acids. Statistical analyses were performed using the Perseus software (Max Planck Institute of Biochemistry, Munich, Germany, version 1.4.0.6). Each sample was analyzed in triplicate to minimize variability and increase the reliability of results and statistical analyses. For each experimental group, only proteins identified in at least 65% of samples were considered. Statistically significant differences were analyzed by Student’s *t*-test and an FDR-adjusted *p*-value (q-value < 0.05).

### 2.6. Functional Enrichment Analyses

Gene Ontology (GO) enrichment analysis using the online bioinformatic tool DAVID version 6.8 [18] was conducted to attribute a biological significance of secretome changes identified in hEDS myofibroblasts. For this purpose, only statistically significant GO terms and clusters of up- and down-regulated differentially expressed proteins (DEPs) with an FDR-adjusted *p*-value < 0.05 and a kappa threshold of 0.7 were included. PANTHER [19] and STRING v.11 [20] biological databases were also employed to respectively recognize protein class and potential protein-protein interaction networks, using a fold enrichment >1.5, FDR < 0.05, and an adjusted *p*-value < 0.05.

### 2.7. Doxycycline Treatment

The effect of doxycycline (doxy) (Sigma Chemicals, #D9891, Milan, Italy) on the organization of FN-ECM was analyzed growing control and hEDS cells up to 72 h in complete MEM supplemented with increasing doses (5, 10, 25, 50, 100 μM) of doxy, refed every 48 h. To explore the organization of COLLI-ECM, control and hEDS cells were grown up to 6 days in complete MEM supplemented with 50 μM AA, refed every 24 h, and 50 and 100 μM doxy, refed every 48 h. To analyze the doxy effect on the organization of TNs-ECM, αvβ3 integrin, Snail1/Slug and α-SMA, both cell types were treated up to 8 days with 50 and 100 μM doxy, refed every 48 h. The organization of FN- COLLI- and TNs-ECM, αvβ3 integrin, Snail1/Slug, and α-SMA cytoskeleton was analyzed by IF, as reported above, every 48 h. In addition, control fibroblasts were grown up to 10 days (6 for COLLI- and TNs-ECM) in the presence of C-CM and hEDS-CM supplemented with 50 μM doxy, refed every 48 h, and analyzed by IF for all the above-mentioned ECM and myofibroblast-associated proteins.

## 3. Results

### 3.1. hEDS-CM Affects ECM Organization of Control Dermal Fibroblasts and Induces Myofibroblast Differentiation

**Figure 1:**
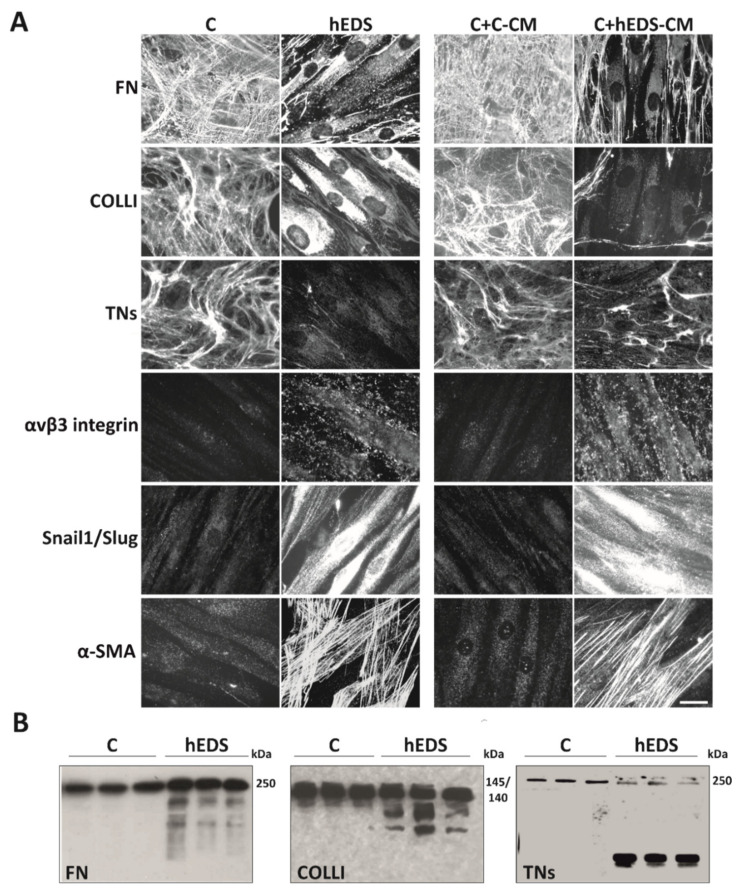
Proteolytic and differentiation potential of hEDS-CM. (**A**) On the left: IF analyses of FN-, COLLI, and TNs-ECM organization, αvβ3 integrin and Snail1/Slug transcription factor expression, and α-SMA cytoskeleton assembly in control (C) and patient (hEDS) dermal fibroblasts grown for 10 days (6 days for TNs and COLLI) in complete MEM. The images are representative of 6 different cell strains for each group. On the right: IF analyses of control dermal fibroblasts grown for 10 days (6 days for TNs and COLLI) in the presence of a pool of CM recovered from six 72 h-grown control (C + C-CM) and six hEDS (C + hEDS-CM) cell strains. Images are representative of three independent experiments. Scale bar: 12 μm. (**B**) WB of 80 µg of proteins recovered from the above-mentioned pooled control and hEDS-CM immunoreacted with anti-human FN Ab, goat anti-human COLLI Ab, and with a mAb recognizing all human isoforms of TNs. WB data represent three technical replicates for each group.

To elucidate the proteolytic and differentiation activity of hEDS-CM, pooled CM from six hEDS and six control fibroblasts were used to verify their effect on the organization of FN-ECM, its αvβ3 integrin receptor, COLLI- and TNs-ECM, and the expression of the myofibroblast-associated proteins Snail1/Slug and α-SMA on control fibroblasts treated up to 10 days and performing IF every 48 h. As shown in Figure 1A, hEDS-CM (C + hEDS-CM), but not control CM (C + C-CM), induced in control cells disorganization of the FN-, COLLI-, and TNs-ECM, organization of distinct αvβ3 integrin patches, especially in focal adhesion sites, expression of Snail1/Slug both in the cytoplasm and nucleus, and well-organized α-SMA cytoskeletal stress fibers. Interestingly, this hEDS-like phenotype conversion of control cells was time-dependent. Indeed, COLLI- and TNs-ECM disorganization was fully evident after 6 days of treatment, whereas for complete FN-ECM disorganization, and αvβ3, Snail1/Slug, and α-SMA induction 10 days were necessary (Figure 1A). At early time points, no significant effect was evident for TNs, whereas for COLLI at 4 days and for FN, αvβ3, Snail1/Slug, and α-SMA at 8 days of culture a partial phenotypic switch was appreciable (Figure S1).

As a further evidence of a possible increased proteolytic activity in hEDS-CM, we analyzed COLLI, FN, and TNs in the pools of control and hEDS-CM by WB. As shown in Figure 1B, the presence of different degradation fragments of all analyzed ECM proteins was identified in hEDS-CM, whereas in controls only the intact form of the proteins was detected. Together, these data strongly suggested that hEDS-CM contain active matrix-remodeling enzymes (e.g., MMPs) and other soluble factors that disturb ECM organization and induce a myofibroblast-like phenotype.

### 3.2. Secretome Analysis of hEDS-CM

To discover potential bioactive molecules involved in ECM disorganization and myofibroblast differentiation of patients’ cells, we achieved LC-MS/MS analyses on CM from each single control and hEDS cell strain used in the previous experiments, after 24 h serum starvation. This proteomic survey recognized a total of 656 proteins (Table S2). After filtering the ones found in at least 65% of samples per group, 112 proteins were identified (Table S2). Following the application of Student’s *t*-test and an FDR-adjusted *p*-value < 0.05, we identified 32 proteins significantly changed in the secretome of hEDS myofibroblasts, 21 of which showed increased levels and 11 a decreased expression (Table 1).

All DEPs were analyzed for their classification by querying protein families PANTHER database (Table S3), which identified an enrichment of different ECM proteins involved in cell-matrix interactions, including COLLs (COL1A1, COL3A1, COL5A2, COL6A3), FN, thrombospondin 2 (THBS2), perlecan (HSPG2), galectin 1 (LGALS1), and matrix-remodeling enzymes such as MMP1 and TIMP2, followed by some endoplasmic reticulum (ER)-resident proteins (PPIB, TXNDC5, P4HB, PDIA3), protease inhibitors (IGFBP3, SERPINH1, SERPING1), cell-adhesion molecules, calcium binding proteins, and metabolite interconversion enzymes (ISLR, TGFBI, NUCB1, CALU, HEXB, ENO1).

GO enrichment analysis was performed for the biological process, molecular function, and cellular component by querying the DAVID database and using an FDR-adjusted *p*-value < 0,05 as filtering cut-off (Table 2 and Table S3).

Functional analysis identified “extracellular matrix organization”, “collagen fibril organization”, “platelet degranulation”, “cell adhesion”, and “extracellular matrix disassembly”, as the most perturbed biological functions. The most enriched GO categories for cellular component were “extracellular region/matrix”, “extracellular exosomes”, followed by “focal adhesion”, “collagen trimer”, and “endoplasmic reticulum-Golgi intermediate compartment” (Table 2 and Table S3).

KEGG pathway analysis and functional annotation clustering identified an enrichment of ECM reorganizing pathways, i.e., ECM-receptor interaction and focal adhesion, followed by PI3K-Akt signaling and maintenance of ER protein folding and cell redox balance (Table S4).

**Figure 2:**
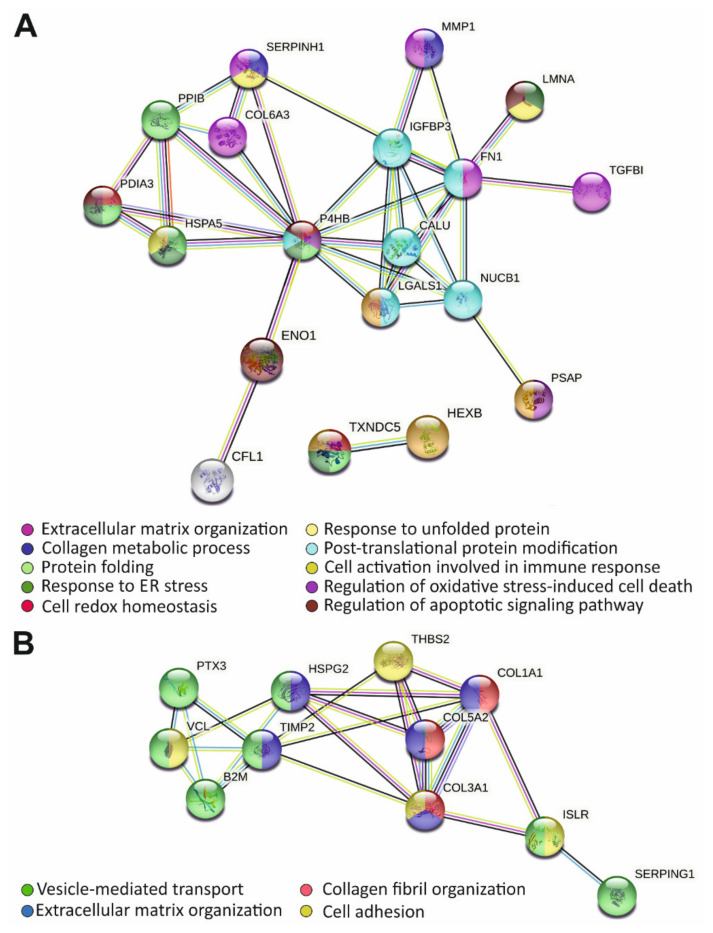
STRING analysis uncovering protein-protein interactions of up- (**A**) and down-regulated (**B**) proteins identified in the secretome of hEDS cells. Each node represents a protein, and each edge represents an interaction including either physical or functional associations. Only interactions with the highest confidence score (0.9) are shown.

Potential protein interaction patterns were investigated by querying the STRING database (last access on 15 October 2021). The most enriched clusters of the 21 up-regulated proteins predicted an enrichment of proteins pertaining to ECM remodeling, collagen metabolic process, and involved in the maintenance of intracellular equilibrium and proteostasis, response to unfolded protein, and ER stress (Figure 2A). Protein-protein interaction networks of the 11 down-regulated proteins highlighted potential hubs with biological functions mainly related to the ECM organization and collagen fibrillogenesis, cell adhesion, and vesicle-mediated transport (Figure 2B).

The main interesting evidence emerged through the secretome analysis was that in hEDS-CM an unbalanced MMPs/TIMPs-mediated ECM proteolysis seems to occur, strongly corroborating the results reported in Figure 1 and prompting to evaluate whether a broad-spectrum inhibition of MMPs might be able to restore in patients’ fibroblasts a correct ECM organization and avoid their differentiation toward myofibroblast-like cells. For this purpose, we opted to use the tetracycline derivative doxycycline (doxy), as its efficiency in the treatment of pathological conditions associated with excessive ECM degradation is well-recognized [21].

### 3.3. Doxycycline Treatment Rescues ECM Organization and Partly Reverts the Myofibroblast-like Phenotype of hEDS Cells

We first excluded significant changes of cell viability in the presence of doxy by treating control and patient cells with increasing doses of the inhibitor from 5 to 100 µM for 48 and 72 h (not shown). Consequently, we performed a preliminary dose-response assessment under these treatment conditions by analyzing the FN-ECM organization by IF. As shown in Figure S2, doxy induced in hEDS cells a gradual assembly of a fibrillar FN network, which started to be significant after 48 h of 25 µM treatment. The FN-ECM induced in hEDS cells after 48 h of treatment with 50 and 100 µM doxy concentrations was almost comparable to that of untreated control fibroblasts, in which doxy did not significantly affect their FN-ECM deposition.

Next, we treated different patient and control cell strains with both these doxy concentrations and analyzed the FN-, COLLI-, TNs-ECM and the expression/organization of αvβ3, Snail1/Slug and α-SMA by IF which were performed every 48 h with up to 8 days of treatment.

**Figure 3:**
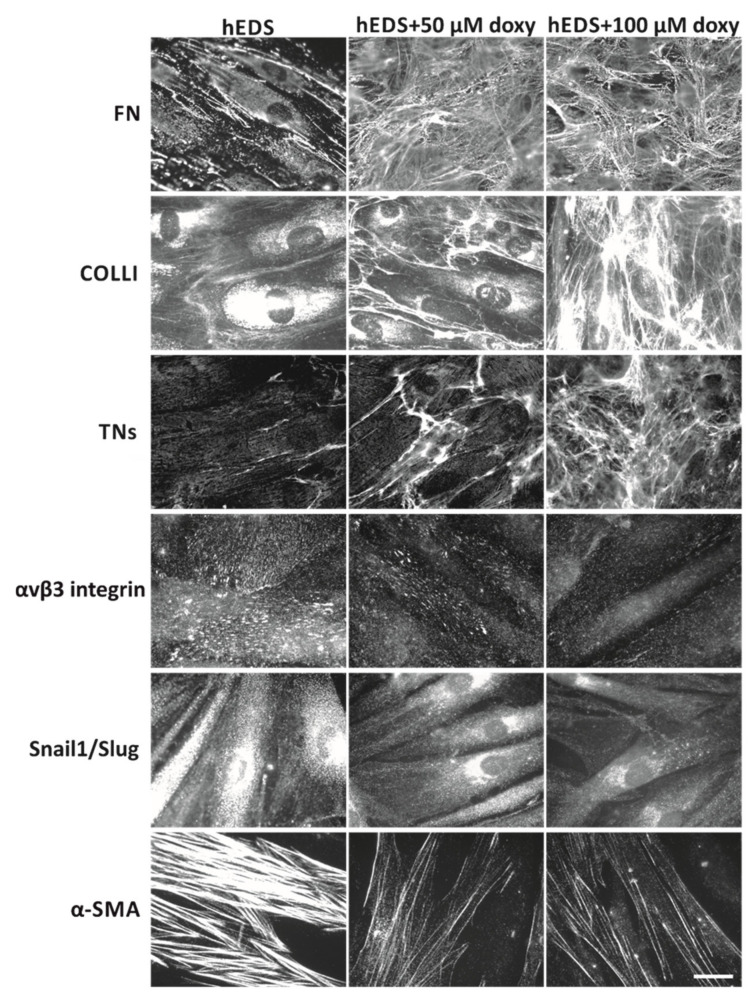
Effect of doxycycline (doxy) on ECM disarray and myofibroblast differentiation in hEDS cells. IF analyses of FN-, COLLI-, and TNs-ECM organization, αvβ3 integrin and Snail1/Slug transcription factor expression, and α-SMA cytoskeleton assembly in hEDS fibroblasts untreated and treated with 50 and 100 µM of doxy in complete MEM for 2 (FN and αvβ3 integrin), 4 (COLLI and TNs), 6 (α-SMA), and 8 (Snail1/Slug) days. The images are representative of 6 different patients’ cell strains. Scale bar: 12 μm.

As shown in Figure 3, in hEDS cells, doxy treatment induced after 48 h, in a dose-dependent manner, not only a control-like FN-ECM but also the disappearance of its alternative αvβ3 integrin receptor on the cell surface. Treatment for 4 days was instead required to observe a significant deposition of TNs and COLLI into the ECM; at the concentration of 100 µM, the fibrillar network of both these proteins was comparable to that of untreated and doxy-treated control fibroblasts (Figure S3). Concerning myofibroblast-associated proteins, 6 and 8 days of treatment were necessary to observe significant disassembly of α-SMA cytoskeletal stress fibers and loss of Snail1/Slug’s nuclear localization. However, compared to treated and untreated control fibroblasts (Figure S3), the doxy-induced myofibroblast dedifferentiation of patients’ cells, at these time reference points and at the maximum concentration tested (100 µM), resulted incomplete, given the presence of residual of α-SMA microfilaments and cytoplasmic Snail1/Slug protein (Figure 3).

**Figure 4:**
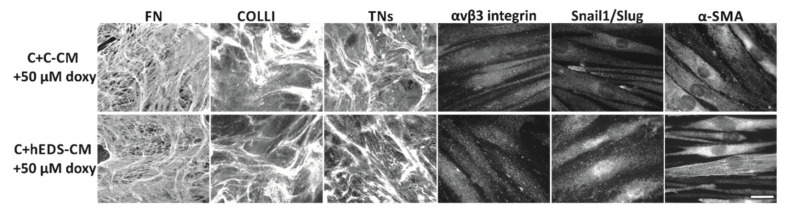
Doxycycline (doxy) abrogates the degradative and differentiation potential of hEDS-CM. IF analyses of FN-, COLLI-, and TNs-ECM organization, αvβ3 integrin and Snail1/Slug transcription factor expression, and α-SMA cytoskeleton assembly in control dermal fibroblasts grown in the presence of a pool of CM, recovered from six 72 h-grown control (C + C-CM) and six hEDS (C + hEDS-CM) cell strains, supplemented with 50 µM of doxy. Images are representative of three independent experiments. Scale bar: 12 μm.

As a further proof of the valuable effect of doxy restoring the ECM organization and reverting the hEDS myofibroblast-like phenotype, we repeated the treatment of control fibroblasts with hEDS and control CM under the same time reference points reported in Figure 1A but supplementing the CM with 50 µM of doxy. As shown in Figure 4, in the presence of doxy, the hEDS-CM was not able to exert its degradative and differentiation activity, since control fibroblasts maintained a proper ECM organization and did not acquire the typical cellular features of patients’ cells.

Together, these findings revealed a potential pathogenetic interplay between an unbalanced MMPs/TIMPs-mediated ECM proteolysis and myofibroblast differentiation and suggest a possible therapeutic application of MMPs inhibition.

## 4. Discussion

The ECM regulates tissue architecture and homeostasis as it provides chemical and mechanical signals to cells modulating their phenotype and responses to coordinate tissue functions. ECM molecules interact with each other creating a functional network that also involves cell–ECM interactions through cell surface receptors that are critical for proper cell and tissue functionality [22]. The importance of the ECM as a multitasking player is underlined by the fact that its perturbation is at the origin of many acquired and inherited human disorders [23,24].

The mechanisms of these ECM-driven diseases include absent factors, aberrant signaling, and disorganization of several structural components [22,25,26]. For instance, the development and progression of various disorders comprising OA, rheumatoid arthritis (RA), fibrotic conditions, and cancer are associated with abnormal ECM remodeling, which drives disease progression by activating specific cell surface receptors and downstream signaling cascades that regulate cell-matrix-interactions [22,27]. In addition, mutations in a plethora of ECM-related genes severely disturb tissue homeostasis causing genetic disorders with variable clinical phenotypes affecting nearly every organ system [23,24]. The molecularly defined forms of EDS, with their 20 ECM-related causal genes currently recognized, are examples of these types of multisystem disorder [1].

hEDS is still believed to be part of this group of inherited disorders, despite its unknown genetic etiology. Recent critical reviews raised the question whether hEDS should be considered to be a single phenotypic entity with an autosomal dominant transmission rather than a spectrum of multifactorial acquired health conditions [28,29,30]. Until large-scale whole genome sequencing and/or association studies will disclose hEDS-associated genetic variants, it is crucial to unravel dysregulated mechanisms underlying abnormal ECM organization in in vitro cell models and their involvement in hEDS, which should allow for the design of novel ECM-based strategies for disease treatment.

Based on our previous [3,4,5,31,32] and current findings, we believe that the driving force in the context of hEDS pathology is a synergistic interplay among ECM perturbation and myofibroblast differentiation. The presence of an unbalanced proteolytic activity in hEDS-CM was proved through the detection of distinct degradation fragments (fs) of COLLI (COLLI-fs), TNs (TNs-fs), and FN (FN-fs) in patients’ cell culture media. In joint inflammatory diseases (e.g., OA and RA), it is well documented that an excessive ECM proteolysis upon cellular stress or tissue injury generates ECM degradation products that act as damage-associated molecular pattern molecules or DAMPs (e.g., FN, tenascin C (TNC), proteoglycans) [33,34,35,36]. These danger molecules promote maladaptive responses by inducing the expression of proinflammatory mediators and cytokines, nitric oxide, along with proteinases (including MMPs) that in turn, degrade further ECM components, creating a degradative and inflammatory feedback loop [37,38,39,40,41]. For instance, high amounts of FN-fs have been detected in cartilage and synovial fluid of OA patients and in vitro stimulations of human articular chondrocytes and synovial fibroblasts with FN-fs recapitulate several features of the pathological phenotype [39,42,43,44]. Similarly, TNC-fs act as endogenous inducers of cartilage ECM degradation by stimulating cytokines and MMPs production through binding with toll-like receptor 4 and integrins including αvβ3 [45,46]. Likewise, degradation products of COLLs alter the cartilage ECM turnover by promoting a catabolic state with the activation of proinflammatory mediators and MMPs [47,48,49]. In line with these findings, it is reasonable to assume that FN-fs, TNs-fs, and COLLI-fs present in the hEDS-CM act as ECM-derived DAMPs. Interestingly, secretome profiling showed higher levels of secreted FN in hEDS compared to control cells, suggesting that FN and its fragments act as one of the main molecular triggers in the disease process. The assumption that these ECM fragments, likely together with others (not yet identified), act as DAMPs is supported by our findings which showed that the treatment of control fibroblasts with hEDS-CM induce an uncontrolled turnover of matrix proteins and consequently a disarray of the ECM. This hEDS-like phenotype conversion of control cells occurred time-dependently. The COLLI- and TNs-ECM disorganization was evident after 6 days of treatment and FN-ECM disorganization was partly appreciable after 8 days, whereas at early time reference points no effect was visible. These observations suggest a feedback mechanism that involves, at least in part, a combination of enhanced levels of MMP1, as indicated by secretome analysis, the previous reported MMP9 [4] likely together with other MMPs not identified by proteomic approach, and damaged ECM fragments present in the hEDS-CM. These latter DAMPs, along with those likely generated by hEDS-CM-derived MMPs on ECM proteins of control fibroblasts, could activate so far uncharacterized cell signaling pathways that stimulate expression of endogenous MMPs with consequent production of further fragments, finally leading to a detrimental feedback loop perpetuating ECM damage. The possible contribution of other proteases and/or inflammatory mediators, either present in the hEDS-CM or induced in control cells, cannot be excluded. The observation that hEDS-CM-treated control fibroblasts exhibited an initial expression of αvβ3 integrin, induction of Snail1/Slug, and a few α-SMA-positive cells at 8 days of culture, strongly suggests that MMPs-mediated FN-ECM degradation and myofibroblast differentiation are likely interconnected through the previously described αvβ3-ILK-Snail1/Slug signaling [4], which rapidly (after two additional days) leads to the full phenotypic switch.

The pivotal role of MMPs as crucial mediators of this vicious cycle was further emphasized by the results obtained treating patients’ cells with the doxy, an FDA-approved antibiotic acting as a nonselective MMP inhibitor. Doxy was able to restore in hEDS cells an appropriate ECM organization alongside a significant attenuation of myofibroblast-like phenotype. Interestingly, and contrary to the effects observed in control cells treated with hEDS-CM, in patients’ cells, doxy first induced the FN-ECM reorganization with concomitant almost complete αvβ3 integrin disappearance (at 2 days), followed by the TNs- and COLLI-ECM reestablishment (at 4 days), whereas for the partial α-SMA and Snail1/Slug disappearance respectively 6 and 8 days of treatment were required. Together, these findings corroborate the hypothesis that MMPs-mediated FN-ECM disorganization, activating an αvβ3 integrin-mediated signaling, is likely an important, but not the only, trigger underlying the hEDS pathogenesis, considering the incomplete myofibroblast dedifferentiation of hEDS cells in the presence of doxy. Besides an unbalanced MMPs/TIMPs secretion in the hEDS-CM, the presence of further pathological contributors is very likely, such as further yet unrecognized secreted factors and other DEPs identified by secretome analysis. Examples of proteins that merit future investigations comprise the glycolytic enzyme α-enolase (ENOA) that acts as a plasminogen receptor mediating activation of plasmin and consequent ECM degradation [50]. Moreover, the pentraxin-related protein 3 (PTX3) and the insulin-like growth factor binding protein 3 (IGFBP3) are both involved in cell proliferation/migration, inflammatory response, and ECM remodeling [51,52], as well as the matricellular protein transforming growth factor beta induced (TGFBI) that binds different ECM components (e.g., FN, COLLs) and participates in cell-ECM interactions through an ανβ3 integrin-mediated mechanism [53]. Interestingly, many of the dysregulated proteins in the hEDS secretome are predicted to be in EVs, which act as modulators of intercellular communications due to their ability to carry a broad range of bioactive molecules, including ECM components, matrix-modifying enzymes, and different RNA species [54]. Therefore, a possible contribution of EVs in the molecular mechanisms underlying disease pathogenesis seems plausible. In this regard, ongoing research is addressed to dissect the secretome into macromolecular components and EVs to thoroughly decipher not only their protein content but also the EV-RNA cargo that may allow the discovery of additional disease-associated bioactive molecules. Among the different RNAs of EVs, miRNAs are particularly intriguing as it is well recognized that they act as crucial regulators of intracellular signaling events related to myofibroblast differentiation and ECM dysregulation [55,56,57].

Concerning MMPs, the finding that the degradative and differentiation potential of hEDS-CM on control fibroblasts is abolished by doxy further strengthens their pathophysiological impact, hence providing promising perspectives for preventing abnormal ECM remodeling and myofibroblast differentiation with a potential therapeutic translation for hEDS patients’ management. Doxy, acting via MMPs inhibition, immunomodulation, and nitric oxide synthase inhibition, has already provided encouraging results, in terms of biochemical and functional improvements including not only pain but also neuroinflammation-associated symptoms (e.g., anxiety and depression), in several human clinical trials for the treatment of OA and RA [21]. Likewise, clinical trials on X-fragile syndrome (FRAXA) with minocycline, another tetracycline derivative, has also shown to markedly ameliorate patients’ behavioral symptoms (e.g., anxiety, mood disorders, sleep disturbance, defects in memory consolidation) [58], many of which are also frequently observed in hEDS [2]. FRAXA is caused by the lack of the FMRP translational repressor that leads to the up-regulation of proteins implicated in synaptic transmission and plasticity including MMP9. The mode of action of minocycline in improving FRAXA neurological symptoms is clarified by its capability to reduce the levels of MMP9 [59]. Interestingly, the genetic interaction between MMP9 and FMRP also occurs in nonneuronal tissues, which might explain the nonneuronal symptoms of FRAXA patients, such as JHM, the hallmark characterizing hEDS, and some orthopedic features (e.g., flat foot and scoliosis).

Based on these observations in other human disorders with a perturbed ECM homeostasis associated with a pro-inflammatory state, in which MMPs inhibition has already demonstrated its efficiency in mitigating disease progression [21], we hypothesize that also in hEDS an MMPs-involving pathogenesis might explain several of the patients’ clinical signs and symptoms ranging from musculoskeletal complaints including pain to neurological issues. Although our promising findings were obtained in vitro and on a limited number of hEDS patients’ cells, we speculate that tetracyclines or other MMPs inhibitors could alleviate, at least in part, the complex patients’ clinical phenotype. However, prior to plan feasibility studies of MMPs inhibition in hEDS patients, thorough translational research is necessary to confirm the real implication of MMPs/TIMPs dysfunction, also considering the highly heterogeneous nature of the ECM composition which might be diversely perturbed at different body and organ sites. This mandatory in vivo research might eventually unravel one (or more than one) specific MMP as major disease driver(s) and could hence allow identifying more reliable compounds that may include the use of specific MMP inhibitors rather than a broad-spectrum approach which was the primary reason for adverse effects observed in some clinical trials [60].

In conclusion, the data presented here provide unprecedented evidence on a putative disease signature that may pave the way to the development of ECM-based targeted therapeutic strategies with a potential benefit for patients’ management.

## Figures and Tables

**Table 1:** Proteins identified by LC-MS/MS analysis in the secretome of hEDS myofibroblasts.

Protein Accession N° ^a^	Protein Name	Protein Description	Fold-Change	FDR Corrected *p*-Value
Up-regulated proteins				
P03956	MMP1	Matrix metalloproteinase 1	53.02	0.0016
P50454	SERPINH1	Serpin family H member 1	6.19	0.002
Q15582	TGFBI	Transforming growth factor beta induced	3.31	0.002
A6XND1	IGFBP3	Insulin-like growth factor binding protein 3	3.12	0.024
Q3BDU5	LMNA	Lamin A/C	2.60	0.003
P02751	FN1	Fibronectin 1	2.44	0.019
P09382	LGALS1	Galectin 1	2.31	0.001
O43852	CALU	Calumenin	2.20	0.012
P06733	ENO1	Enolase 1	2.14	0.042
P11021	HSPA5	Heat shock protein family A (Hsp70) member 5	2.08	0.006
P30101	PDIA3	Protein disulfide isomerase family A member 3	2.08	0.036
Q07954	LRP1	LDL receptor related protein 1	2.07	0.002
F6SYF8	DKK3	Dickkopf WNT signaling pathway inhibitor 3	2.04	0.041
P07686	HEXB	Hexosaminidase subunit beta	1.90	<0.001
P12111	COL6A3	Collagen type VI alpha 3 chain	1.61	0.029
C9JIZ6	PSAP	Prosaposin	1.60	0.002
Q8NBS9	TXNDC5	Thioredoxin domain containing 5	1.54	0.003
P07237	P4HB	Prolyl 4-hydroxylase subunit beta	1.53	0.016
Q02818	NUCB1	Nucleobindin 1	1.52	0.022
P23528	CFL1	Cofilin 1	1.44	0.023
P23284	PPIB	Peptidylprolyl isomerase B	1.40	0.040
Down-regulated proteins				
P35442	THBS2	Thrombospondin 2	0.76	0.008
P02452	COL1A1	Collagen type I alpha 1 chain	0.71	0.010
P98160	HSPG2	Heparan sulfate proteoglycan 2	0.67	0.031
P18206	VCL	Vinculin	0.65	0.014
O14498	ISLR	Immunoglobulin superfamily containing leucine rich repeat	0.64	0.012
P61769	B2M	Beta-2-microglobulin	0.63	0.021
P16035	TIMP2	TIMP metallopeptidase inhibitor 2	0.63	0.015
P26022	PTX3	Pentraxin 3	0.59	0.004
P02461	COL3A1	Collagen type III alpha 1 chain	0.56	0.003
P05155	SERPING1	Serpin family G member 1	0.56	0.012
P05997	COL5A2	Collagen type V alpha 2 chain	0.42	0.001

^a^ Protein accession number according to the UniProtKB knowledgebase database (last access on 15 October 2021).

**Table 2:** The most enriched GO categories related to DEPs identified in the hEDS secretome.

Biological Process		
GO term	Proteins	FDR
GO:0030198~extracellular matrix organization	COL1A1, COL3A1, COL5A2, FN1, COL6A3, TGFBI, HSPG2	0.0003
GO:0030574~collagen catabolic process	COL1A1, COL3A1, MMP1, COL5A2, COL6A3	0.0005
GO:0002576~platelet degranulation	ISLR, PSAP, FN1, SERPING1, VCL	0.0026
GO:0030199~collagen fibril organization	COL1A1, COL3A1, COL5A2, SERPINH1	0.0028
GO:0007155~cell adhesion	COL1A1, ISLR, FN1, COL6A3, TGFBI, THBS2, VCL	0.0073
GO:0022617~extracellular matrix disassembly	MMP1, TIMP2, FN1, HSPG2	0.015
**Molecular Function**		
GO term	Proteins	FDR
GO:0005178~integrin binding	COL3A1, TIMP2, FN1, TGFBI, P4HB	0.0021
GO:0005518~collagen binding	SERPINH1, FN1, TGFBI, PPIB	0.0053
GO:0003756~protein disulfide isomerase activity	PDIA3, P4HB, TXNDC5	0.0094
GO:0005509~calcium ion binding	LRP1, HSPA5, MMP1, CALU, NUCB1, THBS2, HSPG2	0.0143
**Cellular Component**		
GO term	Proteins	FDR
GO:0031012~extracellular matrix	HSPA5, MMP1, FN1, THBS2, HSPG2, COL1A1, COL3A1, LGALS1, LMNA, COL5A2, CFL1, TIMP2, COL6A3, TGFBI, P4HB	1.32 × 10^−15^
GO:0005576~extracellular region	MMP1, IGFBP3, FN1, THBS2, HSPG2, DKK3, COL1A1, COL3A1, ISLR, COL5A2, PSAP, TIMP2, CALU, SERPING1, COL6A3, PTX3, TGFBI, P4HB, B2M, VCL	1.21 × 10^−11^
GO:0005788~endoplasmic reticulum lumen	PDIA3, COL1A1, COL3A1, HSPA5, COL5A2, SERPINH1, COL6A3, P4HB, PPIB, B2M, TXNDC5	1.21 × 10^−11^
GO:0070062~extracellular exosome	PDIA3, HSPA5, HEXB, IGFBP3, FN1, ENO1, HSPG2, ISLR, LGALS1, CFL1, PSAP, TIMP2, SERPINH1, SERPING1, COL6A3, NUCB1, TGFBI, P4HB, PPIB, B2M, VCL, TXNDC5	1.52 × 10^−9^
GO:0005925~focal adhesion	PDIA3, LRP1, HSPA5, CFL1, P4HB, PPIB, B2M, HSPG2, VCL	3.53 × 10^−6^
GO:0005581~collagen trimer	COL1A1, COL3A1, MMP1, COL5A2, SERPINH1, COL6A3	6.36 × 10^−6^
GO:0005793~ER-Golgi intermediate compartment	HSPA5, SERPINH1, FN1, NUCB1, P4HB	5.69 × 10^−5^

## Data Availability

Most data generated or analyzed during this study are included in this published article and its Additional files. Additional data and materials are available from the corresponding author on reasonable request, subject to compliance with our obligations under human research ethics.

## Supplementary Materials

The following are available online at https://www.mdpi.com/article/10.3390/cells10113236/s1, Figure S1: Proteolytic and differentiation potential of hEDS-CM, Figure S2: Doxycycline dose-response evaluation on FN-ECM organization, Figure S3: Doxycycline effect on ECM organization and myofibroblast differentiation in control cells, Table S1: Clinical features of hEDS patients, Table S2: Summary of secretome analysis, Table S3: Protein family classification and Gene Ontology enrichment analyses, Table S4. Top canonical pathways and functional annotation clustering according to DAVID database.

## Abbreviations

AA	Ascorbic acid
α-SMA	Alpha smooth muscle actin
COLLs	Collagens
COLLI-fs	COLLI-fragments
CM	Conditioned media
C-CM	Control cells-derived conditioned media
DAMPs	Damage-associated molecular patterns
DEPs	Differentially expressed proteins
doxy	Doxycycline
ER	Endoplasmic reticulum
MEM	Earle’s modified Eagle medium
ECM	Extracellular matrix
EVs	Extracellular vesicles
FDR	False discovery rate
FN	Fibronectin
FN-fs	FN-fragments
GO	Gene Ontology
HCTD	Heritable connective tissue disorder
hEDS	Hypermobile Ehlers-Danlos syndrome
hEDS-CM	hEDS cells-derived conditioned media
IF	Immunofluorescence microscopy
JHM	Joint hypermobility
LC-MS/MS	Liquid chromatography with tandem mass spectrometry
MMPs	Matrix metalloproteinases
OA	Osteoarthritis
RA	Rheumatoid arthritis
TNs	Tenascins
TNs-fs	TNs-fragments
TIMPs	Tissue inhibitor of metalloproteinases
WB	Western blotting
