# Supplementary material for: Matrix Metalloproteinases Inhibition by Doxycycline Rescues Extracellular Matrix Organization and Partly Reverts Myofibroblast Differentiation in Hypermobile Ehlers-Danlos Syndrome Dermal Fibroblasts: A Potential Therapeutic Target?
Source: Cells. 2021 Nov 19;10(11):3236. doi: 10.3390/cells10113236 (PMC8621259; doi:10.3390/cells10113236)
Supplement: Supplementary file 1 [file cells-10-03236-s001.zip › cells-1462620 - Supplementary Figures 1-3.pdf]

Supplementary Figures

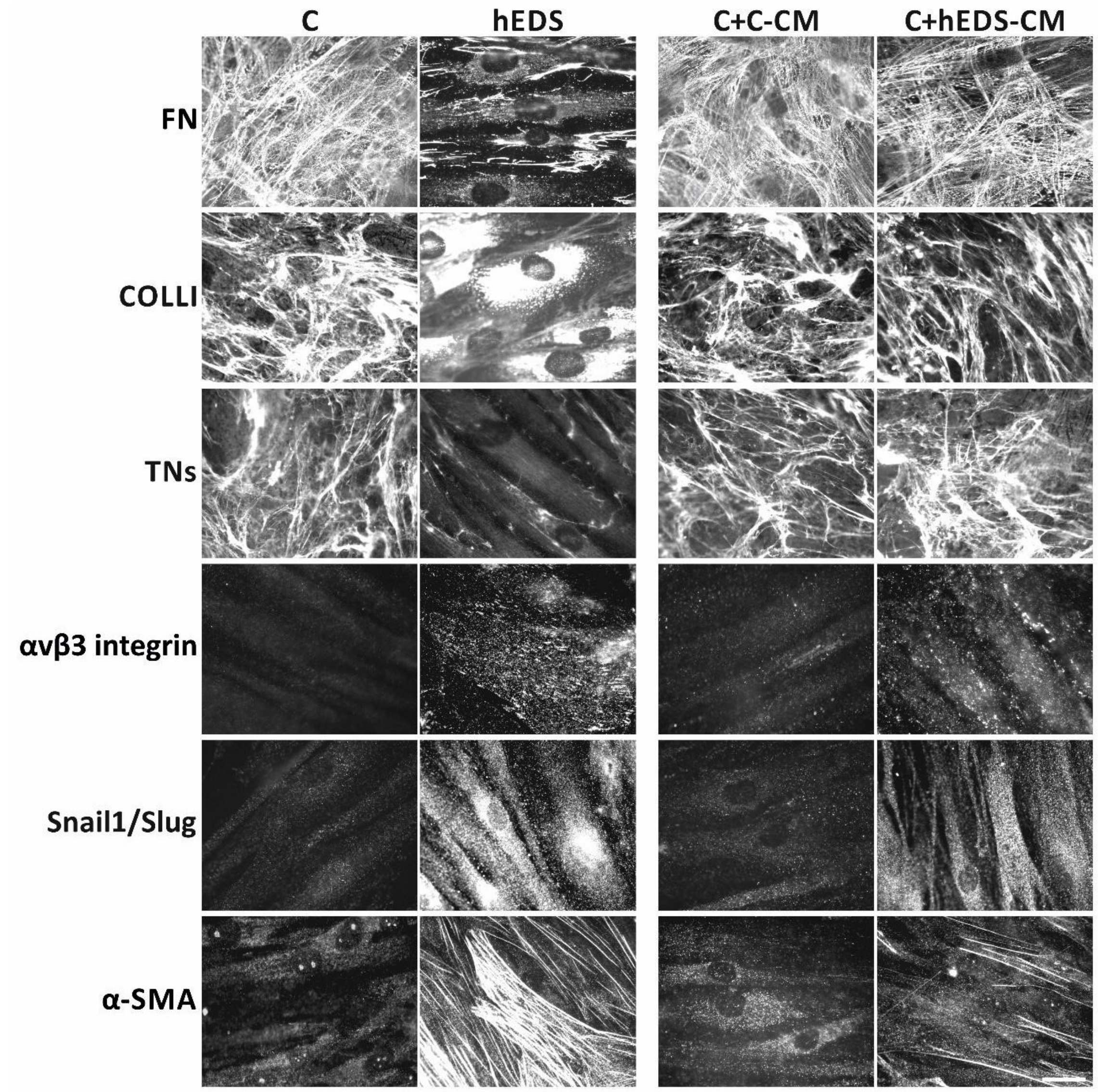

Figure S1. Proteolytic and differentiation potential of hEDS-CM. On the left: IF analyses of FN-, COLLI-, and TNs-ECM organization, $\alpha v\beta 3$ integrin and Snail1/Slug transcription factor expression, and α-SMA cytoskeleton assembly in control (C) and patient (hEDS) dermal fibroblasts grown for 8 days (4 days for TNs and COLLI) in complete MEM. The images are representative of 6 different cell strains for each group. On the right: IF analyses of control dermal fibroblasts grown for 8 days (4 days for TNs and COLLI) in the presence of a pool of CM recovered from six 72 h-grown control (C + C-CM) and six hEDS (C + hEDS-CM) cell strains. Images are representative of three independent experiments. Scale bar: 12 μm.

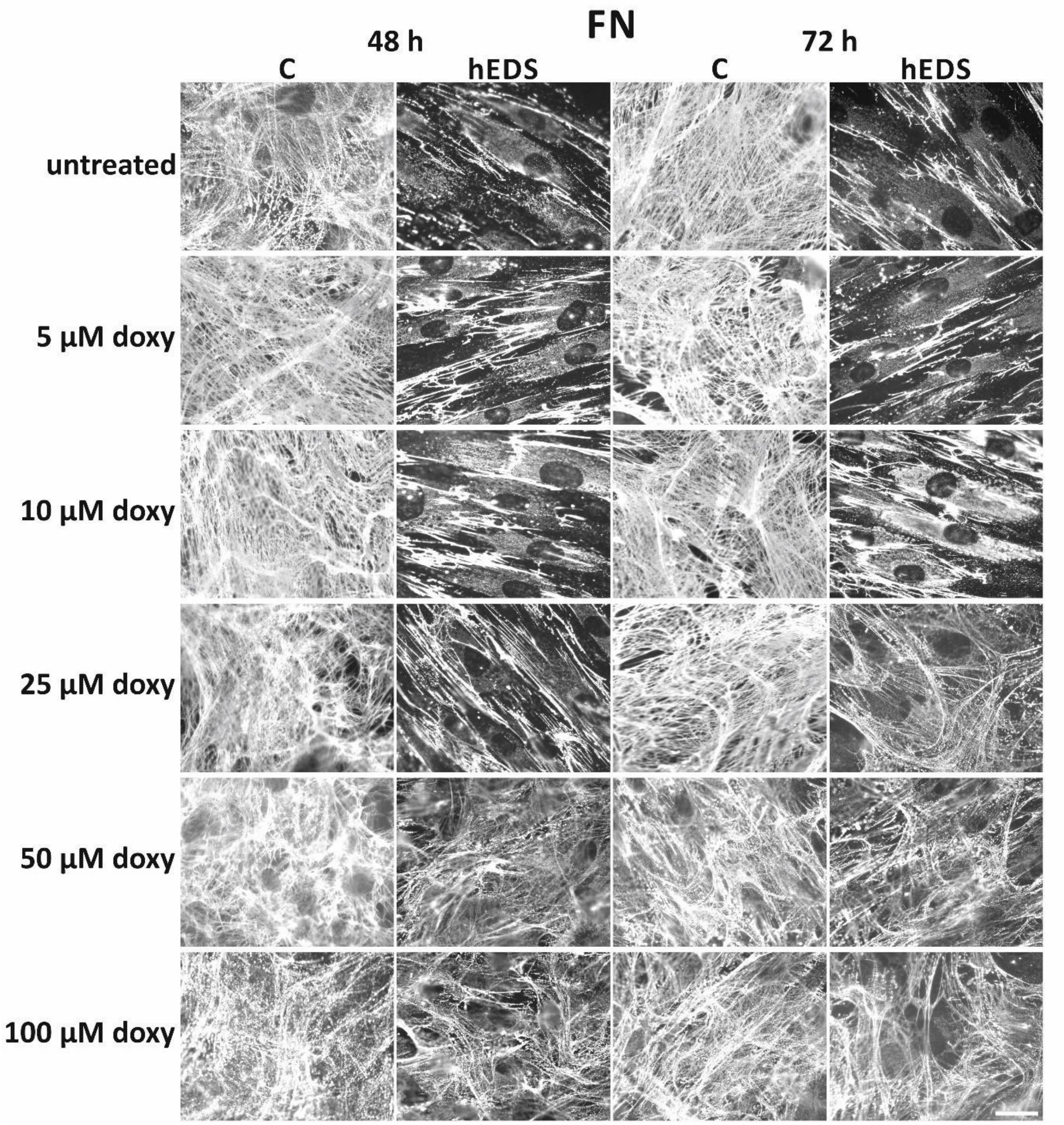

Figure S2. Doxycycline dose-response evaluation on FN-ECM organization in control (C) and hEDS cells grown for 48 and 72 h in complete MEM without or supplemented with increasing doxy concentrations. The images are representative of 6 different cell strains of each group. Scale bar: 12 μm.

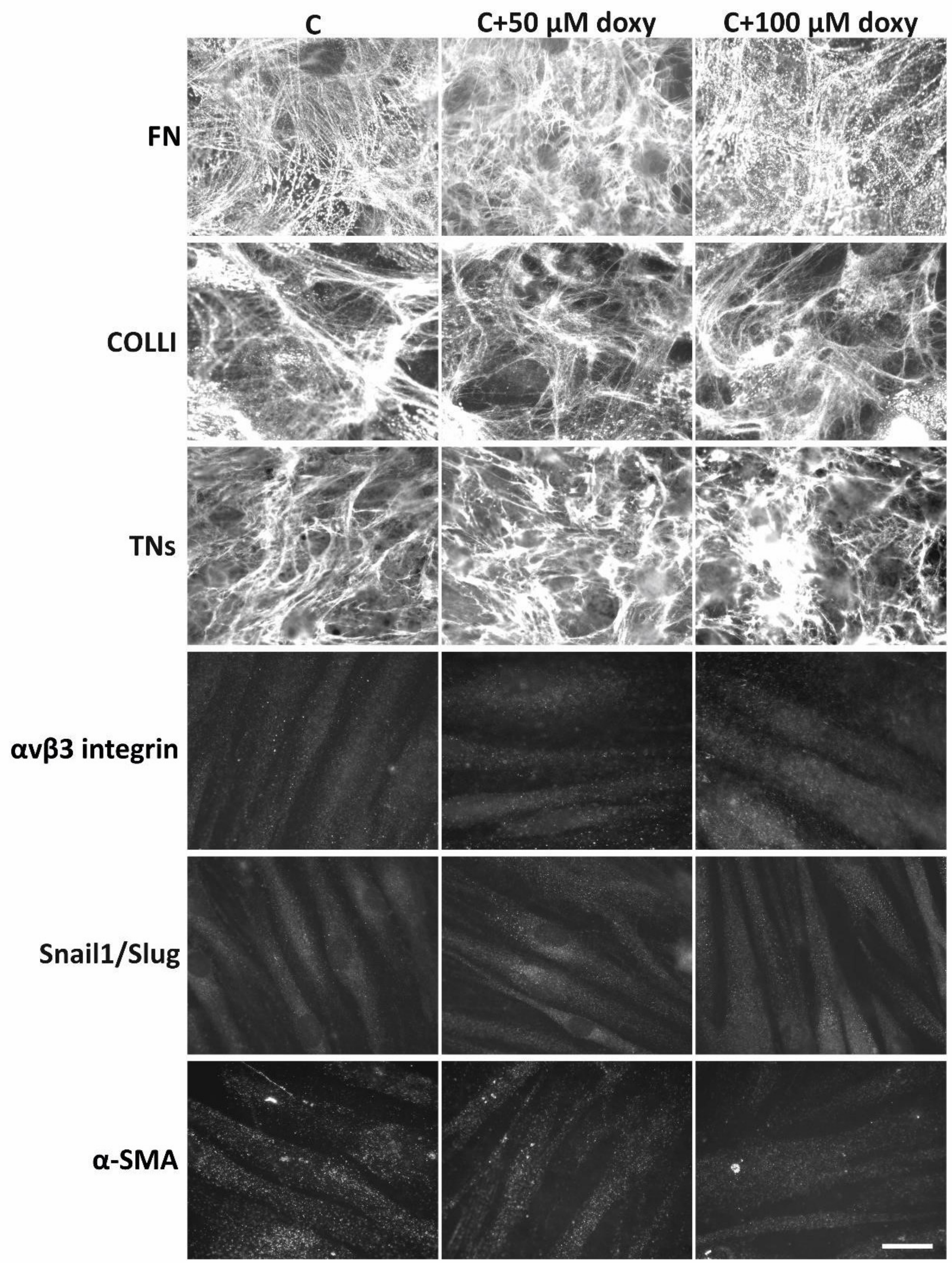

Figure S3. Doxycycline effect on ECM organization and myofibroblast differentiation in control cells. IF analyses of FN-, COLLI-, and TNs-ECM organization, αvβ3 integrin and Snail1/Slug transcription factor expression, and αSMA cytoskeleton assembly in control fibroblasts untreated and treated with 50 and 100 μM of doxy in complete MEM for 2 (FN and αvβ3 integrin), 4 (COLLI and TNs), 6 (α-SMA), and 8 (Snail1/Slug) days. The images are representative of 6 different controls' cell strains. Scale bar: 12 μm.
